# The effect of miR-23b-3p on regulating GH by targeting POU1F1 in Yanbian yellow cattle

**DOI:** 10.1080/10495398.2024.2346808

**Published:** 2024-05-13

**Authors:** Lu Xu, Taihua Jin, Angang Lou, Jiuyang Guan, Xinglin Zhang, Hui Wang, Lizeng Guan

**Affiliations:** a College of Agriculture and Forestry Science, Linyi University, Linyi, China; b Agriculture College, Yanbian University, Yanji, China; c School of Construction Engineering and Mechanics, Yanshan University, Qinhaodao, China

**Keywords:** miR-23b-3p, Yanbian yellow cattle, pituitary cells, GH, POU1F1

## Abstract

This study aimed to evaluate the effect of miR-23b-3p on growth hormone (GH) in pituitary cells of Yanbian yellow cattle. The mRNA and protein levels of GH and miR-23b-3p target genes were measured by real time fluorescence quantitative PCR (qPCR) and Western blot, respectively. The target relationship of miR-23b-3p was validated by double luciferase reporter gene system. The results showed that GH mRNA and protein levels in pituitary cells of Yanbian yellow cattle were significantly lower in the miR-23b-3p-mi group than in the NC group (*P*<0.01), while GH mRNA and protein levels were higher in the miR-23b-3p-in group than in the iNC group (*P*<0.05). The result of bioinformatics analysis and double luciferase reporter gene system validation proved that miR-23b-3p targeted 3′UTR of pituitary specific transcription factor 1 (POU1F1). POU1F1 mRNA and protein levels were lower miR-23b-3p-mi group than in the NC group (*P*<0.01), while POU1F1 mRNA and protein levels were higher in the miR-23b-3p-in group than in the iNC group (*P*<0.01). These results demonstrated that miR-23b-3p could regulate GH expression in pituitary cells by regulating *POU1F1* gene.

## Introduction

Yanbian yellow cattle are an important part of China’s cattle germplasm resources. However, their characteristics such as slow growth and low feed utilization reduce their economic benefits and restrict the development of their rearing scales.^1–3^ Therefore, finding ways to improve the growth rate is the key to accelerating the development of Yanbian yellow cattle breeding industry.

With the development of high-throughput sequencing technology, some genes or non-coding RNAs related to the growth rate of animals have been discovered, such as miRNAs.^4^^,^^5^ MiRNAs are non-coding small RNAs that perform important functions in animals,^6^^,^^7^ by inhibiting the expression of target genes after transcription through binding to the 3′UTR region of the target gene mRNA.^8–11^ Some studies have shown that miRNAs play important roles in regulating the growth and development of animals. For example, Melnik *et al*. found that miRNA in milk exosomes could promote calf growth.^12–14^ Chen *et al* found that miR-PC-86 and miR-PC-263 could regulate the expression of IGF-1R receptor in IPEC-J2 cells and further regulate the growth of porcine muscle cells.^15^ MiRNA-451 could regulate the myogenic differentiation of skeletal muscle in pigs with intrauterine growth restriction.^16^ These studies illustrate that some miRNAs play key regulatory roles in the growth process of animals, so it is a feasible option to explore methods to improve the growth rate of Yanbian yellow cattle from the perspective of miRNAs, especially their effects on GH growth axis (GHAxis), which is closely related to growth rate.

The hypothalamic-pituitary-adrenal (HPA) axis plays an key role in the growth and development of animals.^17–19^ Growth hormone (GH) secreted by the pituitary gland, is one of the most important components of the GHAxis.^20–22^ GH can be transported through the bloodstream with growth hormone binding protein (GHBP) and binds to growth hormone receptors (GHR) in target organs to promote the production of insulin-like growth factors (IGFs). IGFs are then transported to whole body tissues and cells by binding to insulin-like growth factor binding protein (IGFBP) to stimulate the growth and differentiation of bone and chondrocyte and regulate the metabolism of protein, sugar and fat, etc.^23–27^ Research has showed that *GH* gen*e* can be regulated by series miRNA.^28^

Therefore, in this study, the regulation mechanism of miR-23b-3p on GH in pituitary of Yanbian yellow cattle was investigated at the level of pituitary cells in vitro. This study will provide a theoretical basis for further studying on the regulation mechanism of miRNAs on growth and development of animals.

## Materials and methods

### Ethics approval

All procedures with animals received prior approval from the Animal Care and Use Committee of Yanbian University.

### Primary culture of pituitary cells of Yanbian yellow cattle

The pituitary anterior lobe tissues of three Yanbian yellow cattle (5 month old) were separated under aseptic conditions and cut into 1 mm×1 mm×1 mm tissue mass, respectively and then were washed for three times with phosphate-buffered saline (PBS, pH 7.0). The tissue precipitation was transferred to culture flask, and 0.5 mL 0.25% collagenase and trypsin (TaKaRa, Tokyo, Japan) was added to each pituitary tissue and incubated in shaking bed at 37 °C for 25 min. The pituitary cells were filtered with 100 screen mesh. The filtrate was centrifuged at 4 °C and 2,000 rpm for 10 min and the supernatant was discarded. Cells were suspended in DMEM/F12 medium and cultured in 75 cm^2^ culture flask at 37 °C and 5% CO_2_ in an incubator. When growing up to 80% convergence, the cells were digested with 0.25% collagenase. The cells were isolated by differential adherence method and the primary cultured pituitary cells were obtained.

### Transfection efficiency assay of miR-23b-3p on pituitary cells

After the primary culture of Yanbian yellow cattle pituitary cells was established, the pituitary cells were seed in 6-well plate at a density of 2.0 × 10^6^ cells/hole, and transfection test was carried out when the cell confluence reached 60-70%. The pituitary cells were washed with 2 mL PBS for two times before transfection, and 0.8 mL of DMEM/F12 medium was added. Mimics (miR-23b-3p-mi group), mimics reference substance (NC group), inhibitor (miR-23b-3p-in group) and inhibitor reference substance (iNC group) of miR-23b-3p were diluted in 100 µL of DMEM/F12 medium respectively. The miR-23b-3p mimic, inhibitor, NC and inhibitor were purchased from Sangon Biotech Co. (Shanghai, China). The sequences were as follows: miR-23b-3p mimic (5′-AUCACAUUGCCAGGGAUUACC-3′), miR-23b-3p inhibitor (5′-GGUAAUCCCUGGCAAUGUGAU-3′), NC (5′-UCGCAC GGCCAAAAUCCACGU-3′), and iNC (5′-AGGUA CGAAUUCCGGAAGUAC-3′). Then 10 µL transfection reagent Lipofectamine™ 2000 (QIAGEN, Hilden, Germany) was diluted to 100 µL DMEM/F12 culture medium and then mixed with above the diluents at a ratio of 1:1, respectively. After incubation at room temperature for 10 min, 200 µLof the mixture was added into the cell pore of the preconditioned cells, and the final concentration of mimics, mimics control substances, inhibitor and inhibitor control substances of miR-23b-3p was 200 pmol/L, with three replicates per group. After culture for 48 h, the cells were collected and the total RNA was extracted. miR-23b-3p transfection efficiency was measured using by fluorescence quantitative PCR (qPCR).

Total RNA was harvested from the pituitary cells by Trizol reagent and digested with *DNase* I (TaKaRa, Tokyo, Japan) to remove trace DNA contamination. The cDNA synthesis was catalyzed by M-MLV reverse transcriptase (TaKaRa, Tokyo, Japan) using total RNA as a template and the specific stem-loop primer (5′-CTCAACTGGTGTCGTGGAGTCGGCAAT TAGTTGAGCACAAATT-3′) (Qi et al. 2015). Primer Premier 6.0 software was used to design the forward and reverse primers for the PCR amplification of miRNA and U6 gene. The forward and reverse primers of miR-23b-3p were 5′-ATCACATTTGTAGACACG-3′ and 5′-ACCACTCGTGGAGAGC-3′, respectively; The forward and reverse primers for U6 were 5′-CTCGCTTCGGCAGCACA-3′ and 5′-AACGCTTCACGAATTTGCGT-3′, respectively. PCR mixture contained 5 µL of SYBR Green Master Mix (TaKaRa, Tokyo, Japan), 0.5 µL of 10 mmol/l each of primers, 1 µL of cDNA and 3 µL of PCR water. The PCR condition was as follows: 95 °C for 5 min, followed by 40 cycles of 95 °C for 10 s, 56 °C for 20 s, and 72 °C for 20 s. Fluorescence signals were then collected. The transfection efficiency of miR-23b-3p was calculated by the 2^-ΔCT^ method. The formula was as follows: ΔCt = miR-23b-3p Ct-U6 Ct.

### Effect of miR-23b-3p on GH mRNA transcription level in Yanbian yellow cattle pituitary cells

The cDNA synthesize was catalyzed by M-MLV reverse transcriptase (TaKaRa, Tokyo, Japan) using total RNA as template and OligodT-18 as the primer. Primer Premier 6.0 software was used to design upstream and downstream primers for GH gene and β-actin gene in Yanbian yellow cattle. The upstream and downstream primers for GH were: 5′-CTCCAACTGCTGGCTGCCGACAGCTA-3′ and 5′-CGATGTCTGCTGGGCTCGT CC-3′, respectively; The upstream and downstream primers for β-actin were: 5′-CCACGAAACTACCTTCAACTC-3′ and 5′-CCCACGAAACTACCTTCAACTC-3′, respectively. The PCR system consisted of 10 µL of SYBR Green Master Mix, 0.5 µL of 10 mmol/l each of primers, 1 µL of cDNA and 8 µL of sterile water. The qPCR procedure was as follows: 95 °C for 1 min, followed by 35 cycles of 95 °C for 15 s, 56 °C for 15 s, and 72 °C for 35 s. Ffluorescence signals were collected at the end of the extension. The expression level of GH gene was calculated by 2^-ΔCT^ method. The formula was: ΔCt = Ct (GH)- Ct (β-actin).

### Effect of miR-23b-3p on GH protein expression in pituitary cells of Yanbian yellow cattle

According to instructions provided with the Total Protein Extraction Kit (Roche, Basel, Switzerland), the total protein was extracted from cells, and the GH protein was measured by Western blot method. Briefly, 10% polyacrylamide gel electrophoresis (PAGE) separating gel and 5% PAGE concentrating gel were prepared according to the conventional method and placed in a vertical electrophoresis tank. After solidification, the wood comb was removed and the 1×glycine buffer was added into the comb hole, and 50 µg of prepared total protein sample was added for electrophoresis. After electrophoresis, the PAGE gel was transferred to polyvinylidene fluoride (PVDF) membrane (Roche, Basel, Switzerland) with a voltage of 110 V for 60-70 min. Then the PVDF membrane was taken out and stained with Ponceau red to test the effect of transmembrane. The PVDF membrane was washed for several times with 1×TBST (Tris-HCl and tween buffer) to remove dye solution, and then sealed for 2 h at room temperature with 5% skimmed milk powder. The sealed PVDF membrane was placed in a 5 mL centrifugal tube and 2 mL 1:5000 diluted first antibody (rabbit, monoclonal antibody) (Bioss, Beijing, China) agaist GH was added and incubated for 12 h at 4 °C. After incubation, PVDF membrane was washed 3 times with 1×TBST solution for 10 min each time. The washed PVDF membrane was placed in a new 5 mL centrifugal tube and incubated at room temperature for 2 h with 2 mL 1:5000 secondary antibody (mouse monoclonal antibody) (Bioss, Beijing, China) of GH protein. After incubation, the PVDF membrane was washed 3 times with 1×TBST for 5 min each time. Then the PVDF membrane was placed in the luminescent liquid for 1 min and exposed using the gel imaging system. The relative expression level of GH was calculated by comparing with the expression level of β-actin as a reference gene.

### Target gene prediction of miR-23b-3p

Targetscan and RNA hybridization analysis software were used to predict the target relationship between miR-23b-3p and *GH* expressionrelated main genes including growth hormone releasing hormone receptor (*GHRHR*), somatostatin receptor 2 (*SSTR2*), lymphoid enhancer 1 (*LEF1*), pituitary specific transcription factor 1 (*POU1F1*), somatostatin receptor 5 (*SSTR5*) and cyclic adenosine phosphate effector binding protein 1 (*CREB1*).

### Verification of the target relationship between miR-23b-3p and POU1F1

The upstream and downstream 30 bp sequences of *POU1F1* 3′UTR region matched with the seed sequence of miR-23b-3p were synthesized, and meanwhile five bases of *POU1F1* 3′UTR region matching the seed sequence of miR-23b-3p were mutated artificially as a control. The synthetic fragments and mutant fragments were digested by *Xho*I and *Xba*I (TaKaRa, Tokyo, Japan). The recombinant pirGLO-*POU1F1*-3′UTR expression vector and mutant vector were constructed by linking the digested products with the luciferase gene report vector (pirGLO vector), respectively. Mimics (miR-23b-3p-mi group) and mimics control substance (NC group) of miR-23b-3p and the normal or mutant vector of pirGLO-*POU1F1*-3′UTR were diluted to 200 pmol/L respectively, and then co-transfected into Chinese hamster ovary (CHO) cells using transfection reagent Lipofectamine™ 2000. The specific transfection process was as described above. Cells were collected and lysed after incubating for 48 h and luciferase activity was measured using Luciferase Detection Kit (Promega, Wisconsin, USA).

### Effects of miR-23b-3p on the transcriptional level and protein expression of POU1F1 in pituitary cells of Yanbian yellow cattle

The mRNA transcription level and protein expression of *POU1F1* were evaluated by the detection methods for *GH* described above.

### Statistical analyses

All statistical analyses were performed using SPSS 17.0 statistical software (SPSS 17.0, Chicago, IL, USA). One-way ANOVA was performed to examine the difference between treatments. *P* < 0.05 indicated significant difference and *P*<0.01 indicated extremely significant difference.

## Results

### Transfection efficiency assay of miR-23b-3p on pituitary cells

**Figure 1.**
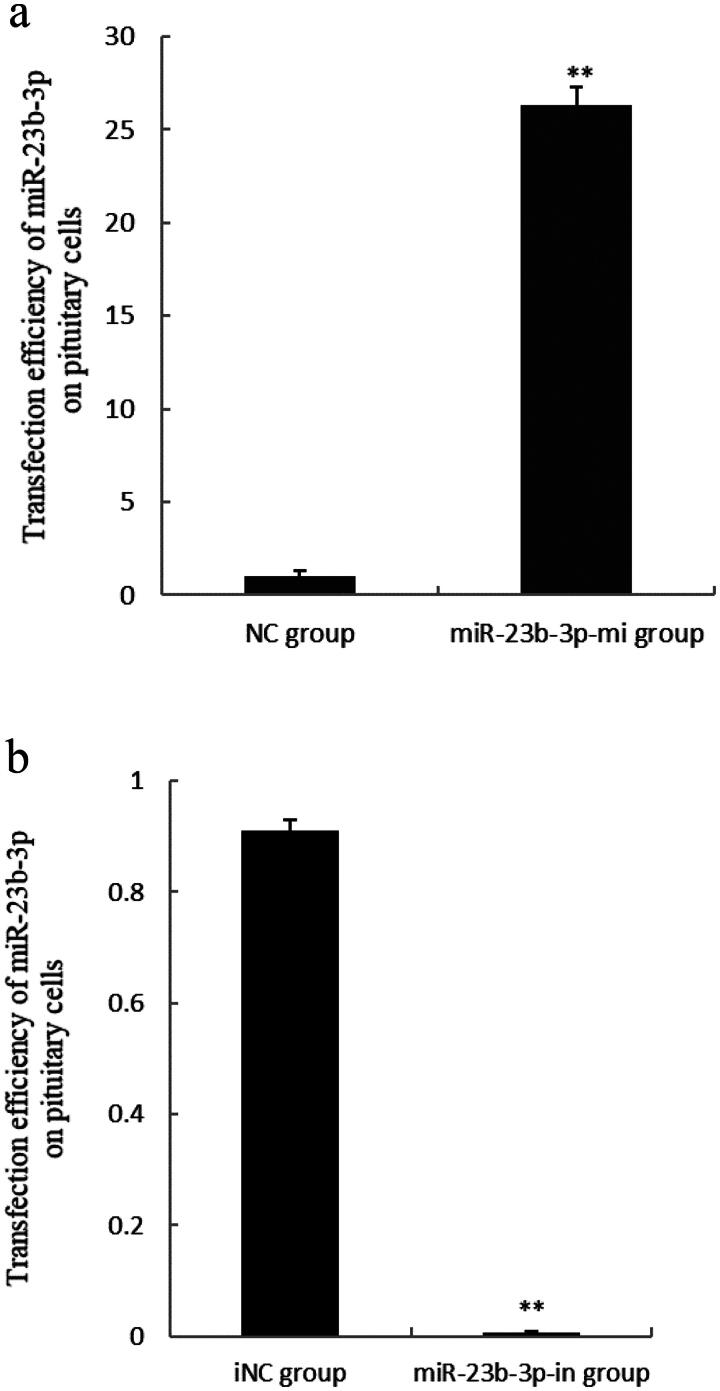
Transfection efficiency assay of miR-23b-3p on pituitary cells of Yanbian yellow cattle. (a) The level of miR-23b-3p in pituitary cells transfected with the mimics (miR-23b-3p group) and mimics control substance (NC group) of miR-23b-3p. Compared with NC group, the column marked by ** showed significant difference (*P*<0.01); (b) The level of miR-23b-3p in pituitary cells transfected with the inhibitor (miR-23b-3p-in group) and inhibitor control substance (iNC group) of miR-23b-3p. U6 was used as an internal reference.

The pituitary cells were transfected with miR-23b-3p mimics and inhibitor, and qPCR was used to detect miR-23b-3p transfection efficiency. The result showed that transfection efficient of miR-23b-3p on pituitary cells was very high (Figure 1a and b).

### Effect of miR-23b-3p on GH mRNA transcription level in pituitary cells of Yanbian yellow cattle

**Figure 2.**
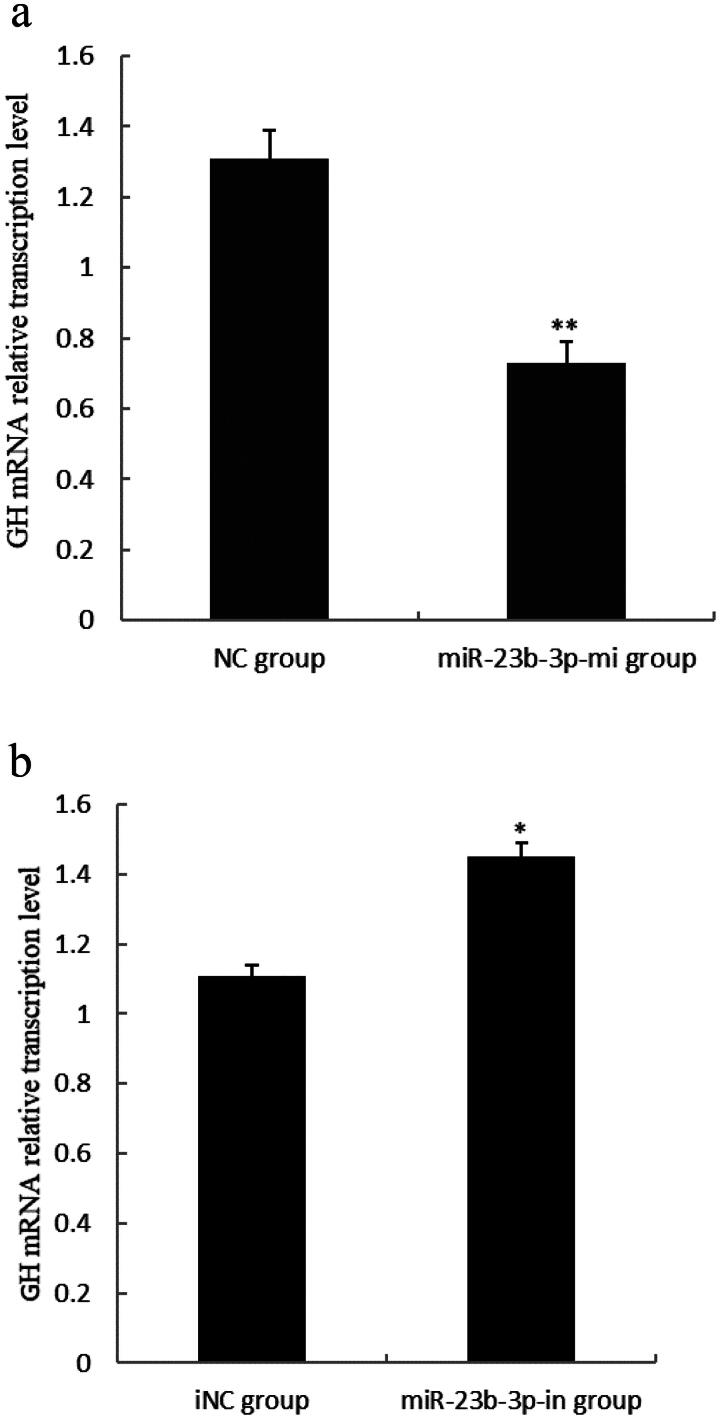
Effect of miR-23b-3p on GH mRNA transcription level in pituitary cells of Yanbian yellow cattle. (a) The relative transcription level of GH mRNA in pituitary cells transfected with miR-23b-3p mimics. Mimics (miR-23b-3p-mi group) and mimics reference substance (NC group) of miR-23b-3p were transfected into pituitary cells of Yanbian yellow cattle, with three replicates in each group. Compared with NC group, the column marking** showed extremely significant difference (*P*<0.01); (b) The relative transcription level of GH mRNA in pituitary cells transfected with miR-23b-3p inhibitor. Inhibitor (miR-23b-3p-in group) and inhibitor reference substance (iNC group) of miR-23b-3p were transfected into pituitary cells of Yanbian yellow cattle, with three replicates in each group. Compared with iNC group, the column marking* showed significant difference (*P*<0.05). β-actin was used as an internal reference.

In order to analyze the effect of miR-23b-3p on pituitary GH mRNA transcription level in Yanbian yellow cattle, the primary cultured pituitary cells of Yanbian yellow cattle were transfected with mimics (miR-23b-3p-mi group), mimics control substance (NC group), inhibitor (miR-23b-3p-in group) and inhibitor control substance (iNC group) of miR-23b-3p. As determined, by qPCR, GH mRNA level in pituitary cells of Yanbian yellow cattle in miR-23b-3p group was extremely significantly lower than in NC group (*P*<0.01) (Figure 2a), while GH mRNA transcription level in pituitary cells of Yanbian yellow cattle in miR-23b-3p-in group was higher than in iNC group (*P*<0.05) (Figure 2b). These results suggested that miR-23b-3p could regulate GH mRNA transcription.

### Effect of miR-23b-3p on GH protein expression level in pituitary cells of Yanbian yellow cattle

**Figure 3.**
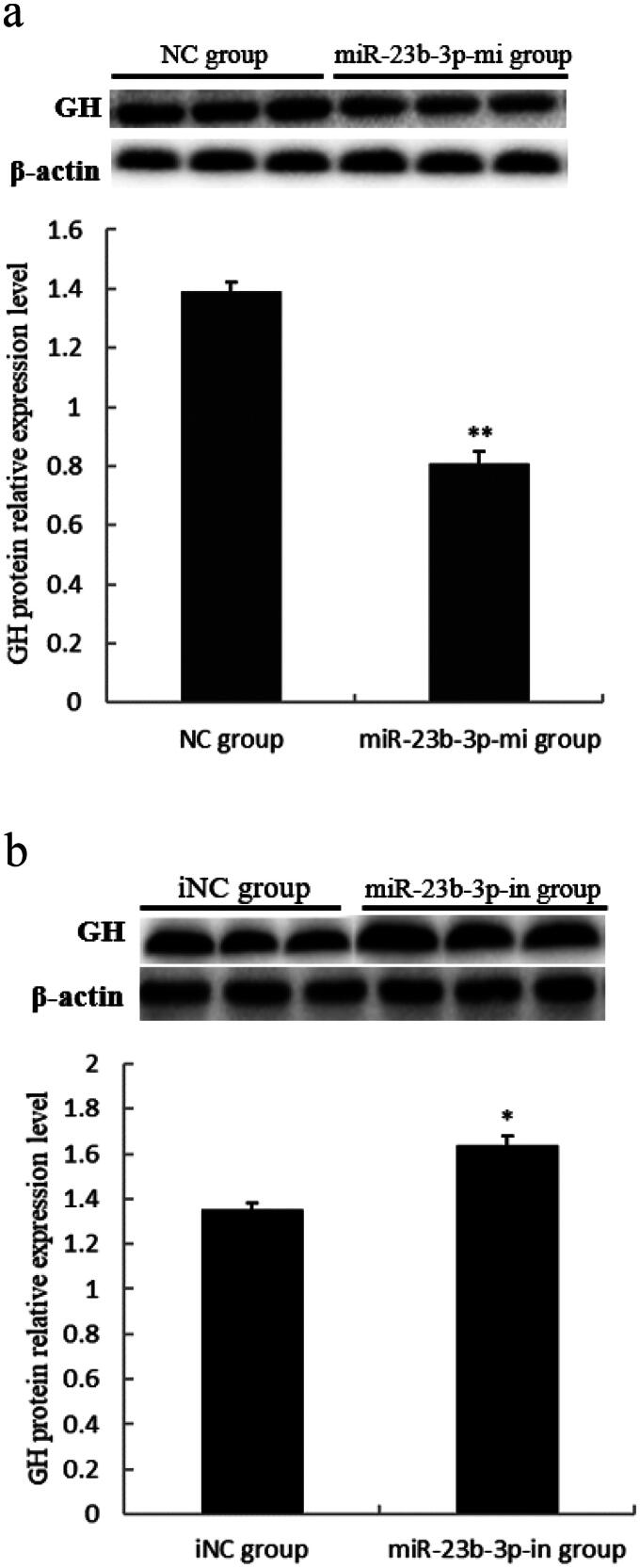
Effect of miR-23b-3p on GH protein expression level in pituitary cells of Yanbian yellow cattle. (a) The relative expression level of GH protein in pituitary cells transfected with miR-23b-3p mimics. Mimics (miR-23b-3p-mi group) and mimics control substance (NC group) of miR-23b-3p were transfected into pituitary cells of Yanbian yellow cattle, with three replicates in each group. Compared with NC group, the column marking** showed extremely significant difference (*P*<0.01); (b) The relative expression level of GH protein in pituitary cells transfected with miR-23b-3p inhibitor. Inhibitor (miR-23b-3p-in group) and inhibitor control substance (iNC group) of miR-23b-3p were transfected into pituitary cells of Yanbian yellow cattle, with three replicates in each group. Compared with iNC group, the column marking* showed significant difference (*P*<0.05). β-actin was used as an internal reference. Electrophoresis of the GH and β-actin gene fragment was from two separate gels.

To further verify the effect of miR-23b-3p on pituitary GH expression in Yanbian yellow cattle, GH protein levels were measured by Western blot based. The results showed that GH protein levels in pituitary cells of Yanbian yellow cattle in miR-23b-3p-mi group were extremely significantly lower than in NC group (*P* < 0.01) (Figure 3a), while GH protein level s in pituitary cells of Yanbian yellow cattle in miR-23b-3p-in group were extremely significantly higher than in iNC group (*P* < 0.05) (Figure 3b). These results suggested that miR-23b-3p could regulate GH protein expression.

### Prediction of the target relationship between miR-23b-3p and GH secretion related genes

**Figure 4.**
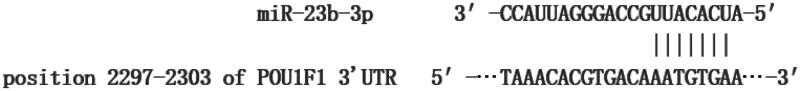
Target relationship between miR-23b-3p and *POU1F1.*

The targetscan and RNA hybrids were used to identify the targets of miR-23b-3p among *GH* secretion-related genes (*GHRHR*, *SSTR2*, *LEF1*, *POU1F1*, *SSTR5* and *CREB1*). The results showed that the seed sequence of miR-23b-3p (UCACAUU) was only complementary to 3′UTR (AATGTGA) of *POU1F1* (Figure 4). It indicated that the target gene of miR-23b-3p was *POU1F1*. In addition, it was concluded that there was not a direct target relationship between miR-23b-3p and *GH* according to the prediction results.

### Verification of the target relationship between miR-23b-3p and POU1F1

**Figure 5.**
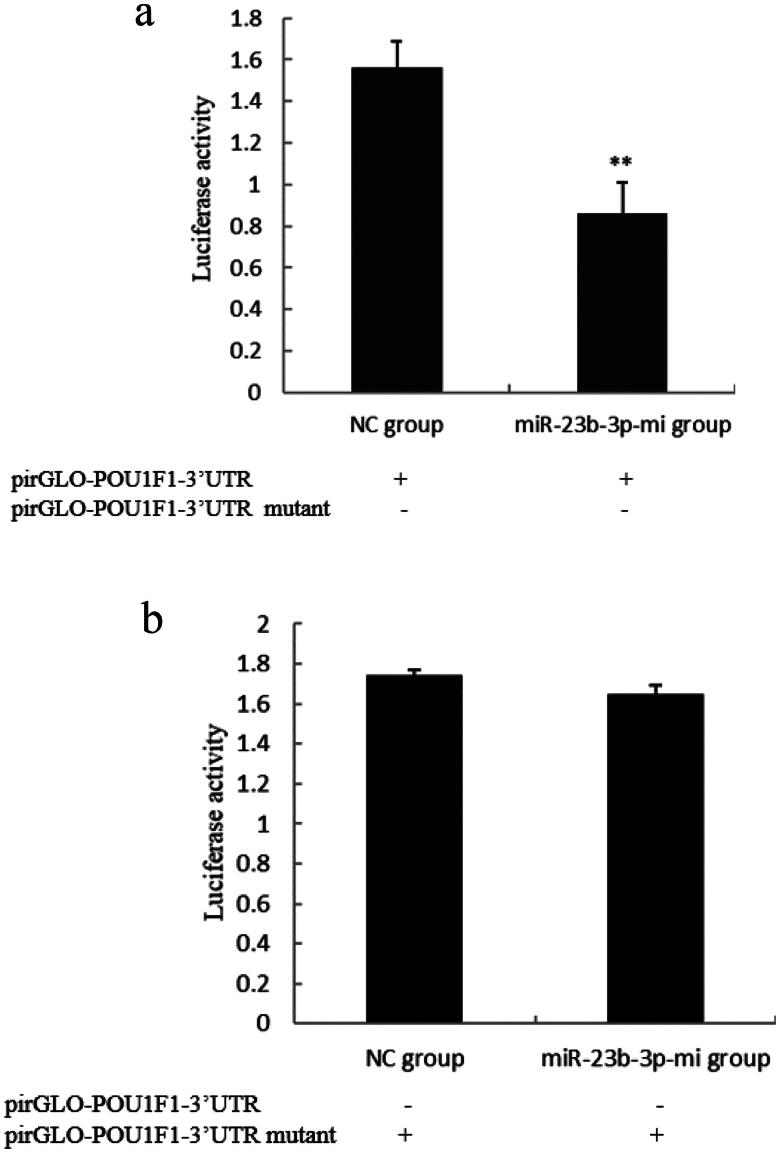
Luciferase activity determination. (a) The changes of luciferase activity after PirGLO-POU1F1-3′UTR normal plasmid co-transfecting with miR-23b-3p mimics (miR-23b-3p-mi group) and mimics control substance (NC group) for 48 h. Compared with NC group, the column marking** showed extremely significant difference (*P* < 0.01); (b) The changes of luciferase activity after PirGLO-POU1F1-3′UTR mutant plasmid co-transfecting with miR-23b-3p mimics (miR-23b-3p-mi group) and mimics control substance (NC group) for 48 h. Compared with NC group, the column without marking**or* showed no significant difference (*P* >0.05).

To further evaluate the relationship between miR-23b-3p and *POU1F1*, pirGLO-*POU1F1*- 3′UTR normal or mutant vectors were co-transfected into CHO cells with miR-23b-3p mimics (miR-23b-3p-mi group) and mimics control substance (NC group), and luciferase activity was observed 48 h later. The results showed that the luciferase activity in pirGLO-*POU1F1*-3′UTR normal plasmid was sig­nificantly decreased by the addition of miR-23b-3p mimics (Figure 5a) (*P*<0.01), but no inhibitory effect of the mimic was observed for the pirGLO-*POU1F1*-3′UTR mutant vector (*P*>0.05) (Figure 5b). Therefore, it could be concluded that miR-23b-3p could bind to and act on the 3′UTR region of POU1F1, supporting the results of the bioinformatics analysis indicating that *POU1F1* is a target of miR-23b-3p.

### Effect of miR-23b-3p on transcription level of POU1F1 mRNA in pituitary cells of Yanbian yellow cattle

**Figure 6.**
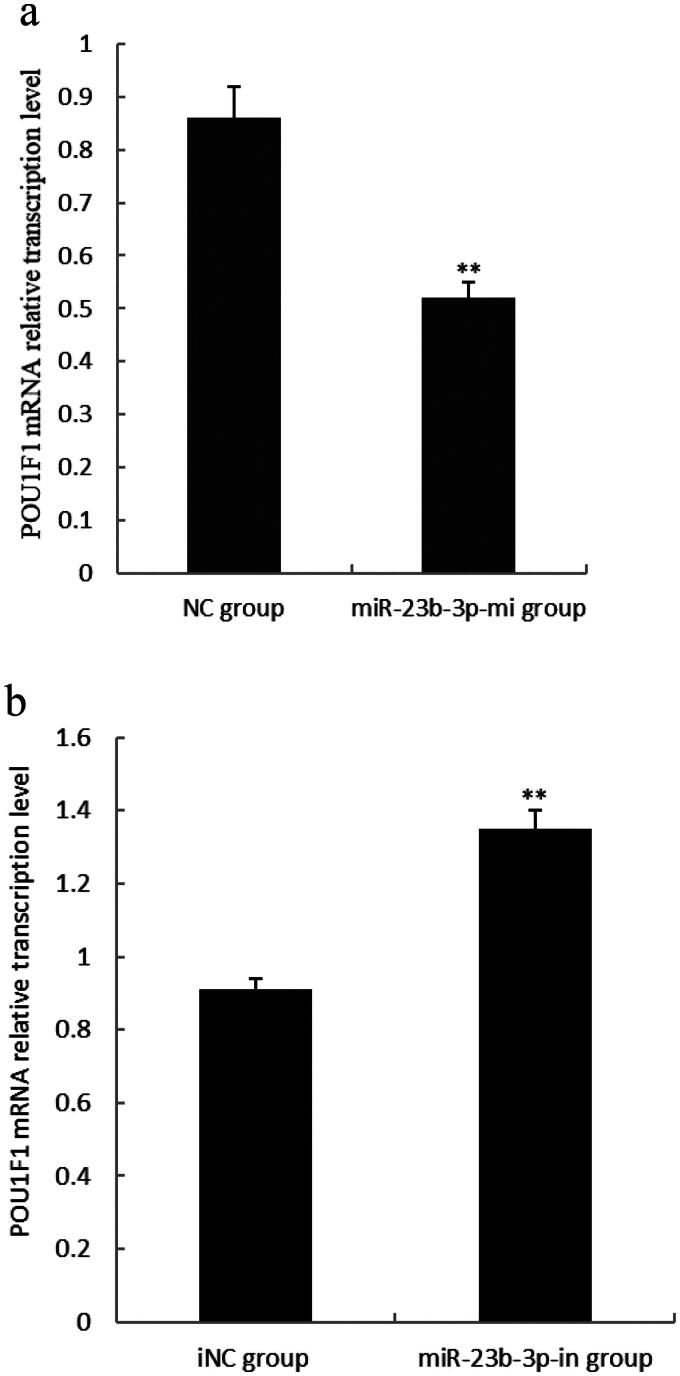
Effect of miR-23b-3p on transcription level of POU1F1 mRNA in pituitary cells of Yanbian yellow cattle. (a) The relative transcription level of POU1F1 mRNA in pituitary cells transfected with miR-23b-3p mimics. Mimics (miR-23b-3p-mi group) and mimics control substance (NC group) of miR-23b-3p were transfected into pituitary cells of Yanbian yellow cattle, with three replicates in each group. Compared with NC group, the column marking** showed extremely significant difference (*P*<0.01); (b) The relative transcription level of POU1F1 mRNA in pituitary cells transfected with miR-23b-3p inhibitor. Inhibitor (miR-23b-3p-in group) and inhibitor control substance (iNC group) of miR-23b-3p were transfected into pituitary cells of Yanbian yellow cattle, with three replicates in each group. Compared with iNC group, the column marking** showed extremely significant difference (*P*<0.01). β-actin was used as an internal reference.

To verify miR-23b-3p could regulate on *POU1F1*, the primary cultured pituitary cells of Yanbian yellow cattle were transfected with mimics (miR-23b-3p group), mimics control substance (NC group), inhibitor (miR-23b-3p-in group) and inhibitor control substance (iNC group) of miR-23b-3p. As determined by qPCR, POU1F1 mRNA levels in the miR-23b-3p-mi group was significantly lower than in the NC group (Figure 6a) (*P* < 0.01), while POU1F1 mRNA levels in the miR-23b-3p-in group was significantly higher than in the iNC group (Figure 6b) (*P* < 0.01). These results suggested that miR-23b-3p could regulate *POU1F1* mRNA transcription.

### Effect of miR-23b-3p on POU1F1 protein expression level in pituitary cells of Yanbian yellow cattle

**Figure 7.**
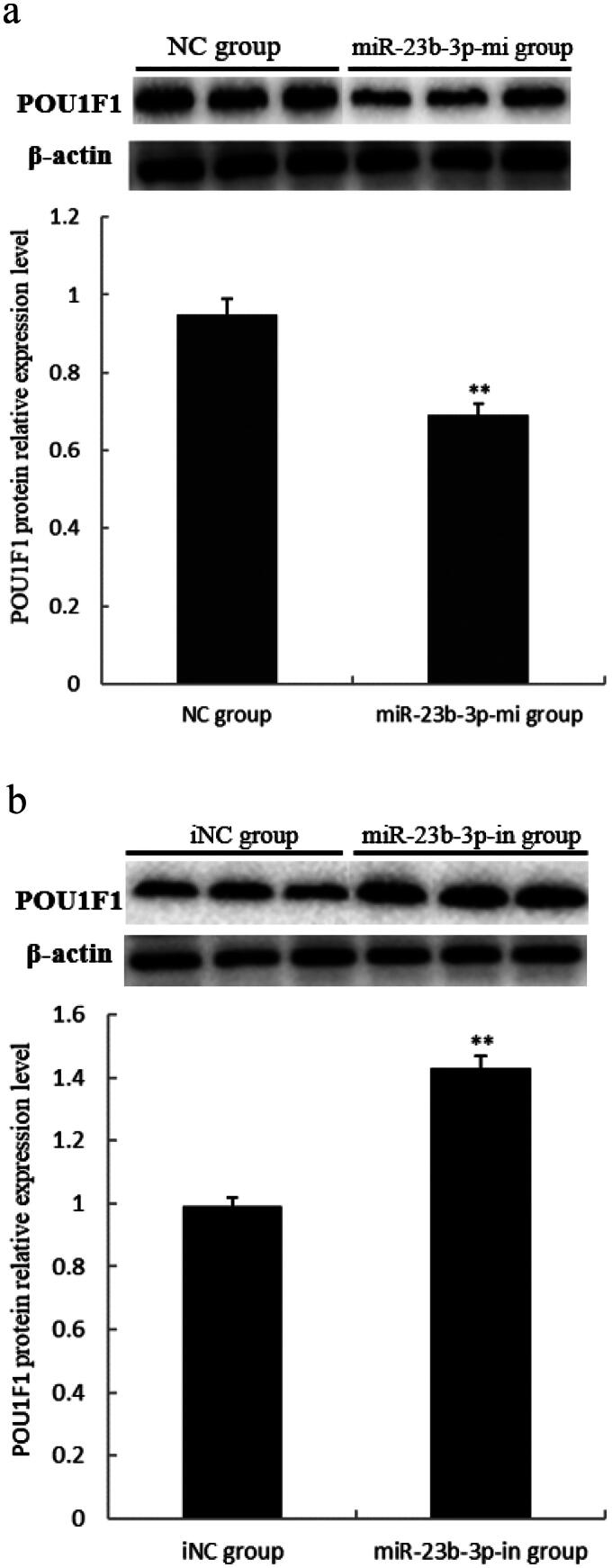
Effect of miR-23b-3p on POU1F1 protein expression level in pituitary cells of Yanbian yellow cattle. (a) The relative expression level of POU1F1 protein in pituitary cells transfected with miR-23b-3p mimics. Mimics (miR-23b-3p-mi group) and mimics control substance (NC group) of miR-23b-3p were transfected into pituitary cells of Yanbian yellow cattle, with three replicates in each group. Compared with NC group, the column marking** showed extremely significant difference (*P*<0.01); (b) The relative expression level of POU1F1 protein in pituitary cells transfected with miR-23b-3p inhibitor. Inhibitor (miR-23b-3p-in group) and inhibitor control substance (iNC group) of miR-23b-3p were transfected into pituitary cells of Yanbian yellow cattle, with three replicates in each group. Compared with iNC group, the column marking** showed significant difference (*P*<0.01). β-actin was used as an internal control. Electrophoresis of the POU1F1 and β-actin gene fragment was from two separate gels.

To further verify the effect of miR-23b-3p on *POU1F1* protein expression in pituitary of Yanbian yellow cattle, POU1F1 protein levels were measured by Western-blot based on the transcription results of *POU1F1* mRNA. The results showed that the *POU1F1* protein levels in the miR-23b-3p-mi group was significantly lower than in the NC group (*P*<0.01) (Figure 7a), while *POU1F1* protein levels in the miR-23b-3p-in group was significantly higher than in the iNC group (Figure 7b) (*P*<0.01). These results suggested that miR-23b-3p could regulate the *POU1F1* protein expression.

## Discussion

Some researches have shown that some miRNAs can regulate animal growth by participating in the regulation of GH expression.^29^^,^^30^ But there are few studies on the regulation of miR-23b-3p on animal growth, especially on the relationship between miR-23b-3p and GH expression. Therefore, in order to determine whether miR-23b-3p is related to GH expression, we first observed the effect of miR-23b-3p on GH mRNA transcription and protein expression at the level of pituitary cells in vitro. The results showed that miR-23b-3p could inhibit *GH* mRNA transcription and protein expression, which indicated that there was a close relationship between miR-23b-3p and *GH*. However, previous researchers have demonstrated that the main function of miRNAs is to negatively regulate the expression of target genes.^31^^,^^32^ But our research confirmed that there was no direct target relationship between GH and miR-23b-3p.Therefore, we concluded that *GH* was not directly regulated by miR-23b-3p, and the target gene of miR-23b-3p needed further verification.

Some research showed that *GHRHR*, *SSTR2*, *LEF1*, *POU1F1*, *SSTR5* and *CREB1* were the main genes related to GH synthesis and secretion.^26^^,^^33–37^ Therefore, through bioinformatics analysis, the target relationship between miR-23b-3p and the 3′UTR region of the above genes was analyzed in this study. According to the prediction and judgment conditions of the target gene, we concluded that miR-23b-3p had a good matching relationship with the 3′UTR region of *POU1F1*. Some studies have shown that POU1F1 protein is a transcription factor specifically expressed in animal pituitary gland, which can promote *GH* transcription and expression and play an important regulatory role in animal growth and development.^38^ By constructing a dual luciferase reporter gene system, we found that although miR-23b-3p could significantly inhibit the luciferase activity of the normal plasmid, it had no effect on the mutant vector. The principle of luciferase reporter gene system is that the expression of luciferase gene in the system is down regulated or inhibited by miRNA directly acting on the 3′UTR region of the gene, which is the most direct evidence of the relationship between miRNA and targets. So, it could be concluded that the 3′UTR of *POU1F1* might be the target of miR-23b-3p.

After determining the relationship between miR-23b-3p and 3′UTR of *POU1F1*, we verified the effect of miR-23b-3p chemical synthesis mimics (miR-23b-3p-mi) and its corresponding inhibitors (miR-23b-3p-in) on *POU1F1* mRNA and protein pituitary cells in vitro. The results showed that miR-23b-3p inhibited the expression of *POU1F1* mRNA and protein at the level of pituitary cells. The results further demonstrated that there was a target relationship between miR-23b-3p and *POU1F1*, and miR-23b-3p had a negative regulatory effect on *POU1F1*. The results further proved that the main function of miRNAs was to negatively regulate the expression of target genes, which was consistent with the previous findings.^39^^,^^40^ In addition, this result also verified the prediction results of bioinformatics. However, the specific regulatory network and mechanism of miR-23b-3p regulating GH expression need further study.

The results demonstrated that miR-23b-3p could regulate GH expression by targeting *POU1F1*. These findings will not only provide a theoretical basis for further study the mechanism of miRNA on regulating animal growth and development, but also provide a new target for the growth and development regulation of Yanbian yellow cattle.
